# Incidence and predictors of HBV functional cure in patients with HIV/HBV coinfection: A retrospective cohort study

**DOI:** 10.3389/fcimb.2023.1130485

**Published:** 2023-02-08

**Authors:** Qingrong Zhang, Hu Wang, Yi Jin, Na Zhou, Lijun Sun, Hao Wu, Haitao Chen, Taiyi Jiang

**Affiliations:** ^1^ School of Public Health (Shenzhen), Sun Yat-sen University, Guangzhou, China; ^2^ Beijing Key Laboratory for HIV/AIDS Research, Clinical and Research Center for Infectious Diseases, Beijing Youan Hospital, Capital Medical University, Beijing, China; ^3^ Medical Department, Beijing Youan Hospital, Capital Medical University, Beijing, China; ^4^ School of Pharmacy, Macau University of Science and Technology, Macau, Macau SAR, China; ^5^ State Key Laboratory of Quality Research in Chinese Medicine, Macau, Macau SAR, China

**Keywords:** chronic hepatitis B, human immunodeficiency virus, HBsAg, antiretroviral therapy, HBeAg

## Abstract

**Background:**

This study was the first to examine the association of baseline clinical factors with the rate of HBsAg clearance in a large retrospective cohort of Chinese patients with HIV/HBV coinfection treated with combination antiretroviral therapy (ART).

**Methods:**

Our retrospective cohort included 431 patients with HIV/HBV coinfection treated with TDF-containing ART. The median follow-up was 6.26 years. Logistic regression was used to investigate the association of baseline variables with HBsAg clearance, and Cox regression was used to investigate the association of baseline variables with time to HBsAg clearance.

**Results:**

The clearance rate of HBsAg in our study was 0.072 (95% CI 0.049~0.101). In the multivariate logistic regression, advanced age (OR=1.1, P=0.007), high CD4 cell count (OR=2.06, P=0.05), and HBeAg positivity (OR=8.00, P=0.009) were significantly associated with the rate of HBsAg clearance. The AUC of the model integrating the above three predictors was 0.811. Similar results were found in the multivariate Cox regression (HR = 1.09, P = 0.038 for age, HR = 1.05, P = 0.012 for CD4 count and HR = 7.00, P = 0.007 for HBeAg).

**Conclusions:**

Long-term TDF-containing ART can lead to HBsAg clearance of 7.2% in Chinese patients with HIV/HBV coinfection. Advanced age, high CD4 cell count, and positive HBeAg at baseline could be regarded as potential predictors and biological markers for HBsAg clearance in patients with HIV/HBV coinfection.

## Introduction

The prevalence of chronic hepatitis B virus (CHB) infection is 8.4% among patients with human immunodeficiency virus (HIV) worldwide (Leumi et al., 2020). Compared with chronic HBV mono-infection, HIV/HBV coinfection accelerates the progression of chronic HBV to liver cirrhosis, hepatocellular carcinoma (HCC), or end-stage liver disease (Cheng et al., 2021). The combination of tenofovir disoproxil fumarate (TDF) with lamivudine (3TC) or emtricitabine (FTC) is the most widely recommended combined antiretroviral therapy (ART) regimen in the treatment of patients with HIV/HBV coinfection (Panel on Guidelines for the Prevention and Treatment of Opportunistic Infections in Adults and Adolescents with HIV, 2022).

Even though more than 90% of patients with HIV/HBV coinfection could ultimately gain HBV DNA suppression after TDF-containing ART (Avihingsanon et al., 2010; Huang et al., 2019; Audsley et al., 2020), they do not achieve the same therapeutic effect of HBV clinical remission as observed in TDF-treated HBV mono-infected patients (Boyd et al., 2017; Kim et al., 2021; Sterling et al., 2021). Furthermore, some studies showed that approximately 10% of patients with HIV/HBV coinfection exhibited HBV viral rebound after achieving an undetectable plasma HBV DNA level (Matthews et al., 2013; Huang et al., 2019). This evidence indicates that virological response, such as HBV DNA suppression, might not be a reliable therapeutic goal in patients with HIV/HBV coinfection. Currently, the clearance of hepatitis B surface antigen (HBsAg), whether with the acquisition of anti-HBs or not, is commonly considered a functional cure and the ultimate therapeutic goal for CHB infection (Yuen et al., 2018).

Previously, we conducted a meta-analysis that showed that the rate of HBsAg clearance in patients with HIV/HBV coinfection treated with TDF-containing regimens was approximately 7.3% (Jiang et al., 2019), which is similar to the finding reported by Boyd et al. (Boyd et al., 2021). Recently, several studies in Caucasians and Thailand found that ethnicity, HBV viral load, CD4 cell count, HBeAg, and quantification of HBsAg were significantly associated with HBsAg clearance among patients with HIV/HBV coinfection treated with long-term TDF-containing ART (Zoutendijk et al., 2012; Matthews et al., 2013; Jiang et al., 2019; Audsley et al., 2020; Bremen et al., 2020). However, the rate of HBsAg clearance in the above study varies greatly, and the significant predictors they found are inconsistent or even contradictory to some extent, possibly because of the differences in the race, sample size, and follow-up time of each cohort.

It is important to understand the potential predictors and biological markers associated with the clearance of HBsAg among patients with HIV/HBV coinfection, which will deepen our understanding of the mechanism of HIV/HBV coinfection and may help physicians make more effective treatment decisions. However, a similar study has not been conducted in mainland China. The current study included the largest cohort of Chinese patients with HIV/HBV coinfection to date and investigated the association of baseline clinical variables with HBsAg clearance.

## Methods

### Characteristics of participants

**Figure 1:**
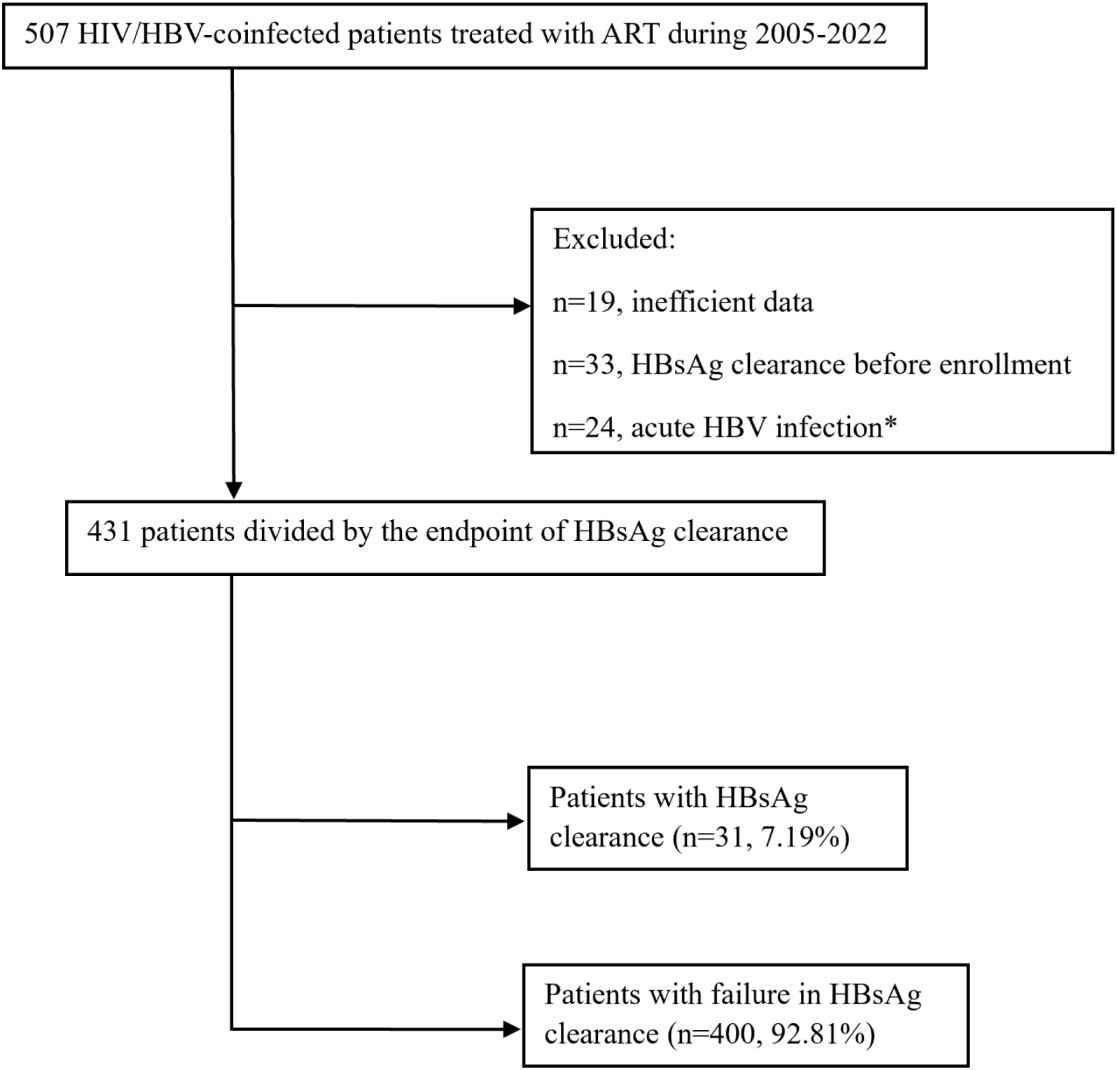
Flowchart of the study population inclusion. *Acute HBV infection was defined as the persistence of HBsAg within 6 months.

Between 2005 and 2022, a total of 507 patients with HIV/HBV coinfection treated with ART at Beijing Youan Hospital were recruited in this study. The inclusion criteria were as follows: 1) enrollment of both patients with HIV and patients with chronic HBV; 2) age >18 years; 3) history of 3TC or 3TC/FTC co-formulated TDF-based antiretroviral therapy (ART); and 4) HBV surface antigen (HBsAg) positivity at baseline. Finally, 431 patients with HIV/HBV coinfection were included in this study (**Figure 1**).

This study was approved by the Ethics Committee of Beijing Youan Hospital. All patients signed an informed consent form before participating in this study.

### Detection of HBV infection

HBV-specific antigens in patient plasma were detected in the clinical laboratory at Youan Hospital using the Elecsys HBsAg Immunoassay (Roche Diagnostics GmbH, Mannheim, Germany) and the immunoassay analyzer Cobase411 (Roche Diagnostics GmbH) according to the manufacturer’s instructions. HBV serological markers were measured using the Beijing Wantaisheng China Pharmaceutical Co., Ltd.

### Plasma HBV DNA monitoring

HBV DNA testing was conducted with real-time polymerase chain reaction (PCR) using the RealART HBV LC PCR kit (Artus GmbH, Hamburg, Germany; with a lower limit of detection [LLOD] of 12 IU/mL or 69.84 copies/mL), Abbott RealTime HBV DNA (Abbott Molecular, Des Plains, Illinois) or Hunan Xiang Sheng Biotechnology Co., Ltd., with an LLOD of 200 copies/mL.

### Liver function tests

Alanine aminotransferase (ALT) and aspartate aminotransferase (AST) levels were determined in the patient’s plasma using ultraviolet-lactate dehydrogenase method test kits (Fortress Diagnostics Limited, United Kingdom).

### Markers of HIV disease progression

Absolute blood CD4^+^ T-cell counts were measured using a FACSCalibur flow cytometer. Viral load was measured by the Amplicor HIV monitor ultrasensitive method with a detection limit of 40 copies/mL of plasma.

### Efficacy measures

Chronic HBV infection was defined as the persistence of HBsAg for at least 6 months. The aspartate aminotransferase (AST)-to-platelet ratio index (APRI) and fibrosis index based on four factors (FIB-4) were used to evaluate liver fibrosis. The endpoint of this study was HBsAg loss, which was defined as having undetectable levels of HBsAg in serum.

### Statistical analyses

Data are expressed as counts and percentages for categorical variables and as medians and interquartile ranges (IQRs) for continuous variables. The Mann−Whitney U test was used to assess the differences in the medians of continuous variables between the two groups when variables were nonnormally distributed. Differences in binary variables were tested using the chi-squared test. Fisher’s exact test was used if 25% of cells had expected counts less than 5. Multivariate logistic regression analyses were performed using a stepwise selection process in which only factors with a P < 0.05 in the univariate logistic regression were included. A P value of 0.05 was used in the stepwise selection procedure for a factor to remain in the model. The discrimination ability of the prediction model derived from multiple logistic regression analysis was quantified by the area under the receiver-operating characteristic curve (AUC), and Delong’s test was used to compare two AUCs of different models (DeLong et al., 1988). Subgroup analyses were conducted to investigate the association of risk factors with HBsAg loss in different age groups (< 35 years and ≥ 35 years) and different HBeAg groups (baseline HBeAg-positive group and baseline HBeAg-negative group). Kaplan−Meier survival curves were depicted to assess the survival probability of HBsAg loss for patients of different ages and HBeAg groups. Univariate and multivariate Cox proportional hazards models were used to investigate the association of baseline factors with time to HBsAg clearance. All analyses were performed using R Studio Version 4.2.1 (R Foundation for Statistical Computing, Vienna, Austria). A two-tailed P < 0.05 was considered statistically significant.

## Results

### Demographic and clinical characteristics of participants

A total of 431 patients were included in this study, and they were divided into the HBsAg clearance group (n=31, 7.19%) and HBsAg nonclearance group (n=400, 92.81%) according to the endpoint (**Figure 1**). The median age of patients with HBsAg clearance was 37, which was significantly higher than that of HBsAg nonclearance patients (37 vs. 32, P=0.016). The majority of participants were male (97.4%). Most of these patients were infected with HIV by MSM transmission (n=418, 85.8%). The median follow-up time for the HBsAg clearance patients was 3.02 years, while for HBsAg nonclearance patients, it was 6.34 (P<0.001). The first-line anti-HBV treatment for most of the patients was TDF-containing ART (n=390, 90.9%). There was a difference in CD4 cell counts between the HBsAg clearance group and the HBsAg nonclearance group (P=0.041). The proportions of HBeAg-positive patients at baseline were 65.2% and 34.1% in HBsAg clearance patients and HBsAg nonclearance patients, respectively (P=0.005). No differences were found for CD4/CD8, AST, ALT, ALP, APRI, and FIB-4 (**Table 1**).

**Table 1:** Baseline clinical characteristics of 431 patients with HIV/HBV coinfection.

	All (n=431)	HBsAg non-clearance at endpoint (n=31)	HBsAg clearance at endpoint (n=400)	P
Age	33 (11)	37 (16)	32 (11)	0.016
Gender				0.728
Male	418 (97.4)	31 (100.0)	387 (97.2)	
Female	11 (2.6)	0	11 (2.8)	
BMI	21.45 (3.65)	21.63 (2.76)	21.40 (3.65)	0.256
Transmission				0.599
MSM	370 (85.8)	28 (90.3)	342 (85.5)	
Not MSM	61 (14.2)	3 (9.7)	58 (14.5)	
Years since admission	6.26 (3.20)	3.02 (2.70)	6.34 (3.00)	<0.001
Treatment regimen				0.754
TDF-containing ART	390 (90.9)	28 (90.3)	362 (91.0)	
Not TDF-containing ART	39 (9.1)	3 (9.7)	36 (9.0)	
HIV VL (10^4)				0.116
≤10^4	110 (40.7)	11 (61.1)	99 (39.3)	
>10^4	160 (59.3)	7 (38.9)	153 (60.7)	
HBV DNA (10^3)				0.222
≤10^3	101 (46.5)	5 (29.4)	96 (48.0)	
>10^3	116 (53.5)	12 (70.6)	104 (52.0)	
CD4 count				0.041
≤100	45 (27.3)	5 (35.7)	40 (26.5)	
(100,350]	75 (45.5)	2 (14.3)	73 (48.3)	
(350,500]	33 (20.0)	5 (35.7)	28 (28.5)	
>500	12 (7.3)	2 (14.3)	10 (6.6)	
CD4/CD8	0.24 (0.28)	0.2 (0.4)	0.24 (0.27)	0.126
≤0.5	141 (85.5)	10 (71.4)	131 (86.8)	
>0.5	24 (14.5)	4 (28.6)	20 (13.2)	
ALT	28.3 (23.9)	33.20 (22.50)	28.25 (24.17)	0.167
AST	27.8 (13.4)	26.40 (11.10)	27.90 (13.42)	0.890
ALP	73.0 (26.7)	71.20 (28.00)	73.2 (26.5)	0.993
Positive HBeAg	123 (36.2)	15 (65.2)	108 (34.1)	0.005
APRI	0.36 (0.32)	0.36 (0.29)	0.36 (0.32)	0.884
FIB-4	0.006 (0.007)	0.004 (0.007)	0.005 (0.006)	0.250

MSM, men who have sex with men.

### Association of baseline variables with HBsAg clearance

**Figure 2:**
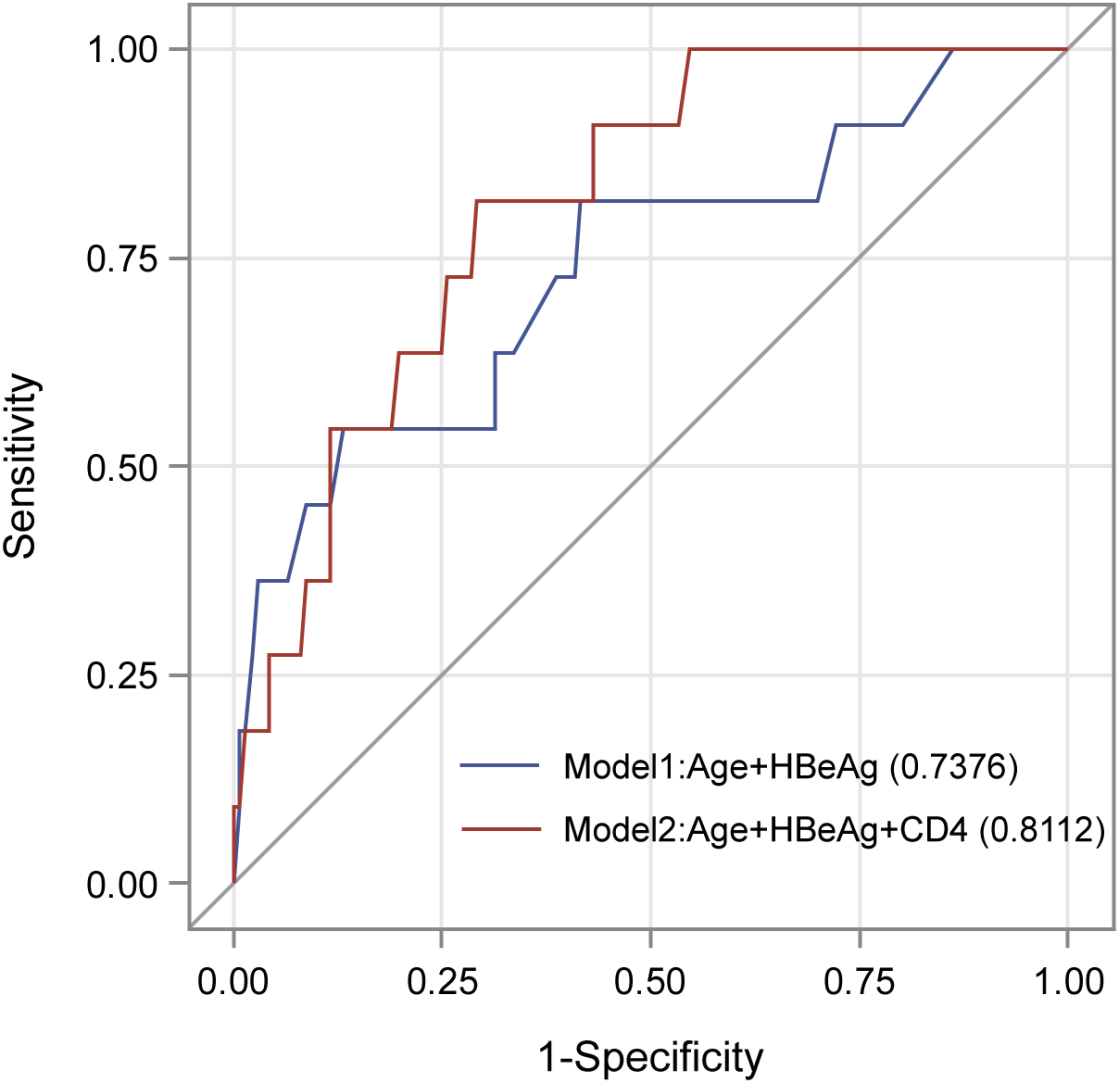
AUC of the two models in the multivariate logistic regression.

We evaluated the association of baseline clinical variables with HBsAg clearance by using logistic regression analyses (**Table 2**). In the univariate logistic regression analyses, increased age (OR=1.05, p = 0.008) and HBeAg positivity (OR=3.63, p = 0.004) were significantly associated with a higher rate of HBsAg clearance. In the multivariate logistic regression analysis, age (OR = 1.08, P=0.018) and HBeAg (OR=6.12, P=0.016) remained significant after adjusting for each other. The AUC of the model consisting of age and HBeAg was 0.738 (**Figure 2**). As CD4 count was another significant variable in **Table 1**, we fit another model by including age, HBeAg, and CD4 count. We found that all three variables remained significant after adjusting for other variables (**Table 2**), and the AUC of the model increased from 0.738 to 0.811 (**Figure 2**). However, the P value for DeLong’s test to compare two AUCs is 0.217, which may be attributed to the different sample sizes of the two models.

**Table 2:** Association of baseline variables with HBsAg clearance in univariate and multivariate logistic regression analyses.

	Univariate logistic		Multivariate logistic 1		Multivariable logistic 2	
	OR (95% CI)	*P*	OR (95% CI)	P	OR (95% CI)	*P*
age	1.05 (1.01-1.09)	0.008	1.08 (1.01-1.15)	0.018	1.10 (1.03~1.18)	0.007
BMI	1.05 (0.9-1.21)	0.551				
Strategy	1.08 (0.31-3.72)	0.906				
HIV VL	0.41 (0.15-1.1)	0.076				
HBV DNA	2.22 (0.75-6.52)	0.149				
CD4 count	1.34 (0.73-2.45)	0.341			2.06 (1.00~4.39)	0.050
CD4/CD8	2.62 (0.75-9.16)	0.131				
HBeAg	3.63 (1.49-8.83)	0.004	6.12 (1.41-26.58)	0.016	8.00 (1.90~46.63)	0.009
APRI	0.96 (0.66-1.40)	0.821				

We further performed a subgroup analysis by dividing all patients into two groups according to age (age < 35 years and age ≥ 35 years) and HBeAg status (HBeAg positive and HBeAg negative). As shown in **Supplementary Table 1**, age was significant in the age < 35 years group, while HBeAg was significant in the age ≥ 35 years group. Age was significant in the HBeAg -positive group, while the CD4 count was significant in the HBeAg -negative group.

### Association of baseline variables with time to HBsAg clearance

By using univariate and multivariate Cox regression, we evaluated the association of baseline variables with time to HBsAg clearance. In the univariate Cox regression, only age (HR=1.05, p = 0.012) and HBeAg status (HR=3.37, p = 0.005) were significantly associated with the time to HBsAg clearance (**Table 3**). Similar to the previous multivariate logistic regression, we fit two models with or without CD4, and we found that age (HR = 1.09, P=0.004), HBeAg (HR = 1.98, P = 0.038) and CD4 count (HR=7.00, P = 0.007) remained significant after adjusting for other variables.

**Table 3:** Association of baseline variables with time to HBsAg clearance in univariate and multivariate Cox regression analyses.

	Univariate Cox		Multivariate Cox 1		Multivariable Cox 2	
	HR (95% CI)	*P*	HR (95% CI)	*P*	HR (95% CI)	*P*
age	1.05 (1.01-1.09)	0.012	1.06 (1.02-1.10)	0.002	1.09 (1.03-1.16)	0.004
BMI	1.05 (0.91-1.22)	0.492				
Strategy	0.52 (0.12-2.19)	0.370				
HIV VL	0.42 (0.16-1.07)	0.070				
HBV DNA	2.24 (0.79-6.36)	0.129				
CD4 count	1.31 (0.74-2.30)	0.349			1.98 (1.04-3.78)	0.038
CD4/CD8	2.36 (0.74-7.54)	0.146				
HBeAg	3.37 (1.43-7.95)	0.005	3.91 (1.65-9.27)	0.002	7.00 (1.68-29.27)	0.007
APRI	1.01 (0.51-1.99)	0.979				

**Figure 3:**
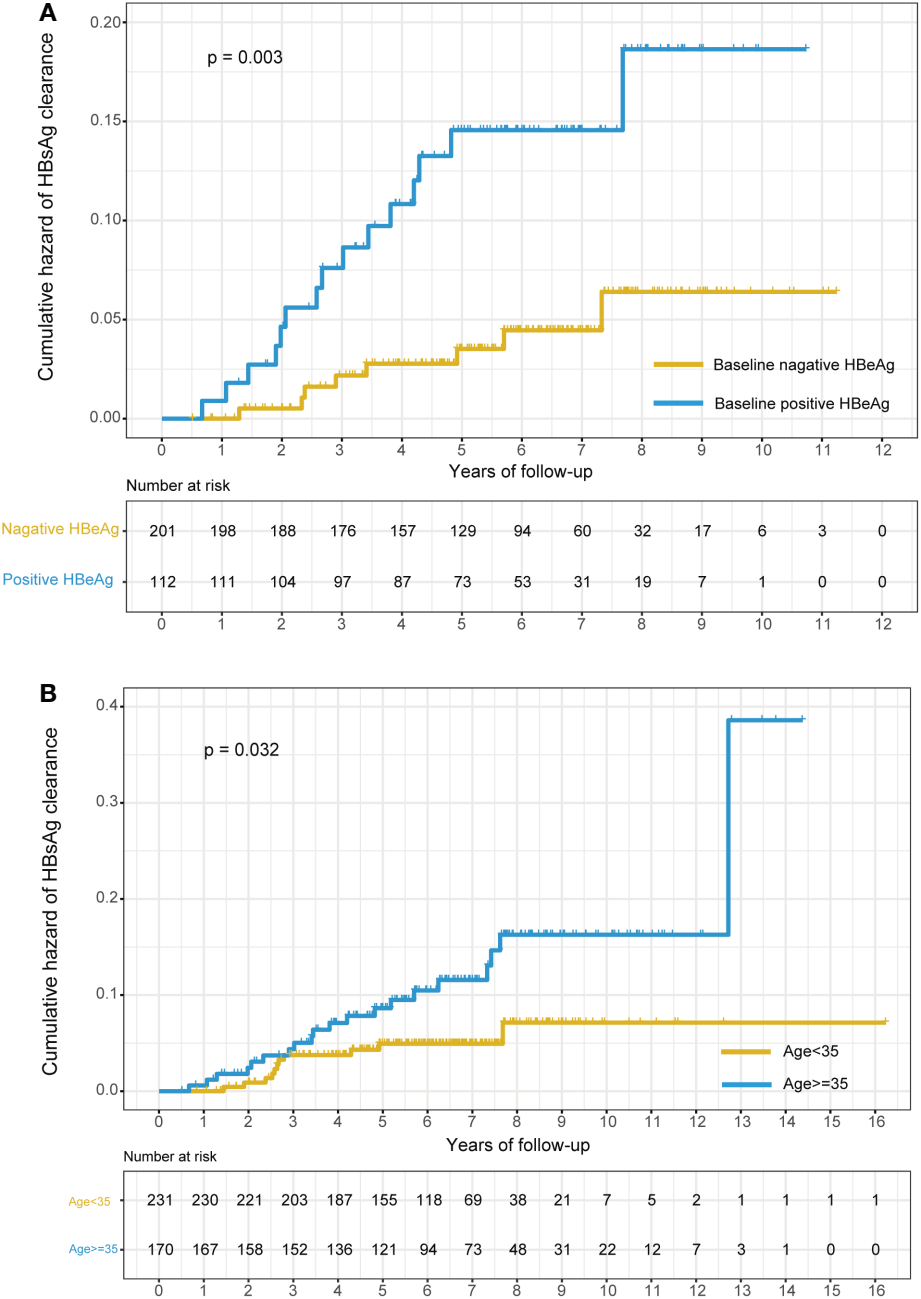
Kaplan–Meier plots of the cumulative hazard of HBsAg clearance stratified by the baseline status of HBeAg and age. **(A)** Stratified by the baseline status of HBeAg (positive HBeAg at baseline or negative HBeAg at baseline); **(B)** stratified by age (age<35 or age>=35).

We also depicted two Kaplan−Meier plots to evaluate the cumulative hazard of HBsAg clearance rate for different age groups (age < 35 years and age ≥ 35 years) and HBeAg status groups (HBeAg positive and HBeAg negative). We found that the cumulative hazard of HBsAg clearance rate in the age ≥ 35 group and HBeAg -positive group was significantly higher than that in the age < 35 group and HBeAg -negative group (P=0.032 for age and P=0.003 for HBeAg, **Figure 3**).

### Plateau effect of HBsAg clearance during follow-up

**Figure 4:**
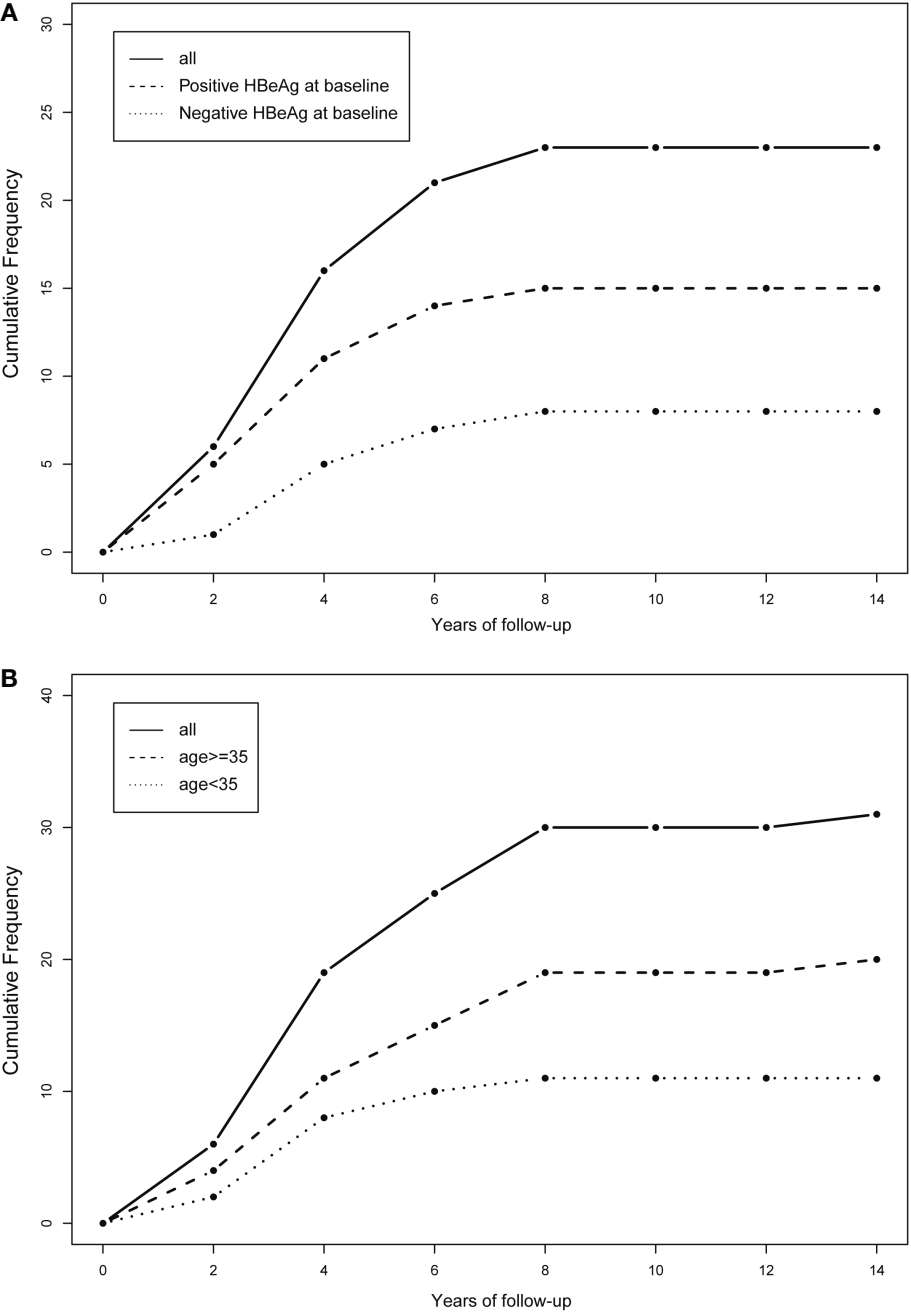
Cumulative frequency of HBsAg clearance stratified by the baseline status of HBeAg and age. **(A)** Stratified by the baseline status of HBeAg (positive HBeAg at baseline or negative HBeAg at baseline); **(B)** stratified by age (age<35 or age>=35).

The cumulative number of HBsAg-loss patients in different age groups and different HBeAg statuses are shown in **Figure 4**. The clearance rate of HBsAg increased sharply in the first few years of ART treatment and then reached a plateau by the 8th year of follow-up. A similar trend was observed in subgroups stratified by age and HBeAg status at baseline.

## Discussion

For the first time, we investigated the association of baseline clinical variables with HBsAg clearance in 431 Chinese patients with HIV/HBV coinfection. We found that age, baseline HBeAg status, and CD4 count were significantly associated with the HBsAg clearance rate. In addition, age, HBeAg status, and CD4 count were also significantly associated with time to HBsAg in the survival analyses.

In previous studies, the rate of HBsAg clearance varied from 4.1% to 21% in patients with HIV/HBV coinfection (Zoutendijk et al., 2012; Hamers et al., 2013; Matthews et al., 2013; Boyd et al., 2015; Boyd et al., 2016; Huang et al., 2019; Audsley et al., 2020; Bremen et al., 2020; Jain et al., 2021). The HBsAg clearance rate in our study (0.072, 95% CI: 0.049~0.101) was very close to that in the American cohort, France cohort, and Netherlands cohort. Similar to our study, these three cohorts all have a long follow-up time of at least 5 years and a large sample size (Zoutendijk et al., 2012; Boyd et al., 2015; Jain et al., 2021). The HBsAg clearance rate in Taiwan’s cohort was slightly lower than that in our study (Huang et al., 2019), which may be explained by the fact that some patients in the Taiwan cohort were treated with lamivudine (FTC) monotherapy, while TDF/FTC combination therapy has a better effect on the clearance of HBsAg than FTC monotherapy (Gantner et al., 2019; Hawkins et al., 2022). The distribution of HBV genotypes varies in different areas. In China, the major prevalent genotypes are B and C, and different genotypes also determine the cure of HBV after treatment. Therefore, larger clinical studies are needed for further confirmation.

Our study found that the rate of HBsAg clearance increased sharply in the first few years of TDF-containing ART and then reached a plateau by 8 years of follow-up. The plateau of HBsAg clearance was also observed after 6 years of TDF-containing ART in an Australian study (Audsley et al., 2020). However, more studies have reported that the plateau occurred earlier, such as 24 weeks, one year, or three years after TDF-containing ART (Zoutendijk et al., 2012; Matthews et al., 2013; Boyd et al., 2015). This difference can be attributed to the fact that these studies analyzed the change in quantitative HBsAg rather than HBsAg clearance observed in our study. Another explanation was that some of these reported studies had shorter follow-up, and patients in their study usually had a history of antiretroviral drug use.

We found that advanced age was more likely to lead to HBsAg clearance after long-term TDF-containing ART (OR = 1.10, p = 0.007; HR = 1.09, p = 0.038). Most studies of patients with HIV/HBV coinfection have reported that age had no impact on the occurrence of HBsAg clearance (Zoutendijk et al., 2012; Boyd et al., 2015; Audsley et al., 2020; Bremen et al., 2020). We speculate that one of the reasons may be that our cohort has a median age of 33 years, which is evidently younger than cohorts with a median age of more than 40 years. Younger age always means greater potential in virus control. This finding was supported by a chronic HBV monoinfection study in which they found that advanced age was associated with a higher rate of HBsAg clearance (Tai et al., 2010).

There is no surprise that we found that a high CD4 cell count and HBeAg -positive status at baseline were conducive to HBsAg clearance after long-term TDF-containing ART. Netherlandish and Sub-Saharan African studies showed that long-term TDF-containing ART led to a significant decrease in HBsAg in HBeAg-positive patients with HIV/HBV coinfection with high CD4 cell counts (Zoutendijk et al., 2012; Boyd et al., 2016). The sub-Saharan African team also demonstrated that a high-level CD4 cell count at baseline is associated with faster HBsAg declines (Maylin et al., 2012). The HBeAg -positive status could be suggested as a surrogate marker for HBV viral load usually accompanied by high HBV DNA in patients with HIV/HBV coinfection (Li et al., 2016), which ultimately results in immune activation and restoration. The HBeAg -positive stage generates less HBsAg than the HBeAg -negative stage because the chromosome integration level caused by HBV double -stranded linear DNA (dslDNA) becomes low (Bill and Summers, 2004; Wu et al., 2022). Mutations in the precore region that occur over time in chronic untreated HBV infection lead to viral mutants that do not produce HBeAg and therefore HBeAg-negative disease, which is associated with periods of high viral replication and necro-inflammatory activity in the liver.

Our study does have some limitations due to the nature of the retrospective study. Some medical records were partly incomplete, such as HBV DNA load, HBV genotype, and ART-adherence data. Despite the above limitation, this study is one of the largest and longest cohorts of HIV/HBV coinfection in China. We set strict inclusion criteria to ensure that all subjects included were chronic HBV patients.

In conclusion, our study found that long-term TDF-containing ART successfully leads to a 7.2% functional cure in 431 patients with HIV/HBV coinfection. Furthermore, we found that advanced age, high CD4 cell count, and positive HBeAg at baseline were significantly associated with a higher rate of HBsAg clearance in patients with HIV/HBV coinfection after long-term TDF-containing ART.

## Data availability statement

The original contributions presented in the study are included in the article/**Supplementary Material**. Further inquiries can be directed to the corresponding author.

## Ethics statement

The studies involving human participants were reviewed and approved by the ethics committee of Beijing Youan Hospital. The patients/participants provided their written informed consent to participate in this study.

## Author contributions

HC, and TJ contributed to conception and design of the study. QZ, NZ, HWa, LS, and HWu extracted data and assessed the methodological quality of included studies. QZ performed the statistical analysis. QZ and YJ wrote the first draft of the manuscript. All authors contributed to manuscript revision and approved the submitted version.
