# Supplementary material for: Incidence and predictors of HBV functional cure in patients with HIV/HBV coinfection: A retrospective cohort study
Source: Front Cell Infect Microbiol. 2023 Feb 8;13:1130485. doi: 10.3389/fcimb.2023.1130485 (PMC9944431; doi:10.3389/fcimb.2023.1130485)
Supplement: Supplementary file 1 [file Table_1.docx]

Supplementary Material

Incidence and Predictors of HBV Functional Cure in HIV/HBV Co-Infected Patients: A Retrospective Cohort Study

# Supplementary Tables

**Supplementary Table 1** Multivariable logistic regression analysis stratified by age and the baseline status of HBeAg

|  | B | Wald | OR_with_CI | P |
| --- | --- | --- | --- | --- |
| Age<35 (n=249) | | | | |
| age | -0.033 | 0.044 | 0.97(0.71~1.37) | 0.834 |
| HBeAg | 0.245 | 0.044 | 1.28(0.12~14.37) | 0.833 |
| CD4 count | 1.778 | 5.791 | 5.92(1.72~37.73) | 0.016 |
| Age>=35 (n=180) | | | | |
| age | 0.102 | 3.06 | 1.11(0.99~1.26) | 0.080 |
| HBeAg | 3.235 | 6.65 | 25.41(3.2~630.05) | 0.010 |
| CD4 count | 0.009 | 0 | 1.01(0.28~3.28) | 0.988 |
| Positive HbeAg (n=123) | | | | |
| age | 0.116 | 6.498 | 1.12(1.03~1.24) | 0.011 |
| CD4 count | 0.361 | 0.571 | 1.43(0.55~3.79) | 0.450 |
| Negative HbeAg (n=217) | | | | |
| age | 0.044 | 0.497 | 1.05(0.91~1.18) | 0.481 |
| CD4 count | 1.665 | 4.797 | 5.28(1.39~33.76) | 0.029 |
